# Astragaloside IV inhibits inflammation caused by influenza virus via reactive oxygen species/NOD‐like receptor thermal protein domain associated protein 3/Caspase‐1 signaling pathway

**DOI:** 10.1002/iid3.1309

**Published:** 2024-06-11

**Authors:** Xiaoli Huang, Yifan Zhou, Yi Li, Ting Wang, Yandong Chen, Yuanhong Zhou, Xiaolin Zhou, Qiang Liu

**Affiliations:** ^1^ Department of Infectious Diseases, The First College of Clinical Medical Science China Three Gorges University & Yichang Central People's Hospital Yichang China; ^2^ Central Laboratory, The First College of Clinical Medical Science China Three Gorges University & Yichang Central People's Hospital Yichang China; ^3^ Department of Cardio‐Thoracic Surgery, The First College of Clinical Medical Science China Three Gorges University & Yichang Central People's Hospital Yichang China

**Keywords:** astragaloside IV, Caspase‐1, inflammation, influenza virus, NOD‐like receptor thermal protein domain associated protein 3, reactive oxygen species

## Abstract

**Background:**

Astragaloside IV (AS‐IV) is the most active monomer in the traditional Chinese herbal medicine *Radix Astragali*, which has a wide range of antiviral, anti‐inflammatory, and antifibrosis pharmacological effects, and shows protective effects in acute lung injury.

**Methods:**

This study utilized the immunofluorescence, flow cytometry, enzyme‐linked immunosorbent assay, quantitative reverse transcription‐polymerase chain reaction, western blot, and hematoxylin and eosin staining methods to investigate the mechanism of AS‐IV in reducing viral pneumonia caused by influenza A virus in A549 cells and BALB/c mice.

**Results:**

The results showed that AS‐IV suppressed reactive oxygen species production in influenza virus‐infected A549 cells in a dose‐dependent manner, and subsequently inhibited the activation of nucleotide‐binding oligomerization domain‐like receptor thermal protein domain associated protein 3 inflammasome and Caspase‐1, decreased interleukin (IL) ‐1β and IL‐18 secretion. In BALB/c mice infected with Poly (I:C), oral administration of AS‐IV can significantly reduce Poly (I:C)‐induced acute pneumonia and lung pathological injury.

**Conclusions:**

AS‐IV alleviates the inflammatory response induced by influenza virus in vitro and lung flammation and structural damage caused by poly (I:C) in vivo.

## Graphical abstract

(1) Astragaloside IV (AS‐IV) can inhibit reactive oxygen species production induced by influenza virus. (2) AS‐IV inhibits the nucleotide‐binding oligomerization domain‐like receptor thermal protein domain associated protein 3/Caspase‐1 signaling pathway induced by influenza virus. (3) AS‐IV can alleviate severe pneumonia induced by Poly(I:C) infection in mice.

## 1 INTRODUCTION

Influenza virus is a major respiratory pathogen that remains the world's most deadly communicable disease.^1^ Studies have shown that the prognosis of viral pneumonia caused by influenza virus is related to the interaction between the virus and the host.^2^, ^3^, ^4^ In mild cases, the hosts can effectively remove pathogens and result to a good prognosis.^2^ However, in severe cases, the violent inflammatory response causes extensive lung tissue damage and hypoxemia which may progress to acute respiratory distress syndrome. As a result, the mortality rate for severe influenza infection ranges from 10% (seasonal influenza)^3^ to 65% (pandemic influenza).^4^ Early antiviral treatment can improve the prognosis of influenza patients. But Dobso et al. found that antiviral treatment alone could not effectively reduce the mortality of severe influenza patients.^5^ Herold et al. further confirmed that the high mortality rate of severe influenza is related to the excessive inflammatory response of the host lung.^2^ Thus, excessive host inflammatory response to influenza virus is the main cause of death in patients with severe influenza.

Nucleotide‐binding oligomerization domain‐like receptor thermal protein domain associated protein 3 (NLRP3) inflammasome is a high molecular weight polyprotein complex whose formation is strictly regulated by initiation and activation signals.^6^ The NLRP3 inflammasome plays a key regulatory role in the innate immune system, recognizing signals from a variety of microorganisms (such as bacteria and viruses), both endogenous and exogenous (crystalline particles, and so on).^6^ After the NLRP3 inflammasome is activated, it can activate the downstream effector Caspase‐1 to mediate pyroptosis. Tate et al. found that the delaying administration of NLPR3 inhibitors in the early stage of influenza virus infection could significantly improve the survival rate of mice and reduce lung injury.^7^ Ong et al. suggest that controlling the activation of the NLRP3 inflammasome at a reasonable level is necessary to maintain homeostasis.^8^ Reactive oxygen species (ROS) is a key factor regulating NLRP3 activation.^9^ Heid et al. have shown that increased ROS can activate NLRP3 inflammasome‐mediated cell damage.^10^ Further, increased ROS can also promote the spread of viruses between cells and increase the body's susceptibility to respiratory viruses.^11^, ^12^, ^13^

*Radix Astragali*, the root of *Astragalus membranaceus* var. *mongholicus* (Bunge) P. K. Hsiao is one of the most popular herbs with multiple functions such as anti‐inflammatory^14^ and antiviral effects.^15^ The study of Liang et al. showed that *R. Astragali* injection significantly inhibited the proliferation of H1N1 and improved the survival rate of cells infected with the H1N1 virus.^16^ Khan et al. demonstrated that aqueous extract of *R. Astragali* has antiviral activity and can be used to treat avian influenza virus infection.^17^ In addition, *R. Astragali* has been shown to possess antihepatitis B virus activities both in vitro and in viro^18^ and exhibits therapeutic effects on myocarditis caused by the Coxsackie virus.^19^ Astragaloside IV (AS‐IV) is the main active component of *R. Astragali*, which has strong antioxidant, antifibrosis, anti‐inflammatory, and antiviral effects.^20^ AS‐IV can reduce lung injury caused by various pathological factors by reducing ROS.^21^, ^22^ AS‐IV can improve pulmonary fibrosis induced by bleomycin‐induced pulmonary fibrosis by inhibiting ROS.^22^, ^23^ AS‐IV alleviates the progression of Chronic Obstructive Pulmonary Disease by inhibiting the production of ROS and inflammatory factors.^24^

Recently, Zhang et al. reported that in H1N1 infection, AS‐IV attenuates the secretion of the inflammatory factor interleukin (IL);‐1β by enhancing autophagy.^25^ However, the specific mechanism of action of AS‐IV in influenza virus infection remains unclear. In many diseases, AS‐IV reduces inflammation by inhibiting the production of ROS. Therefore, in this study, we combined the antioxidant properties of AS‐IV to further explore the possible mechanism of AS‐IV in influenza viral pneumonia and develop effective anti‐influenza viral pneumonia drugs from the perspective of reducing viral inflammation and body damage.

## 2 MATERIAL AND METHODS

### 2.1 Cells and viruses

Human lung carcinoma cell lines (A549 cells) (ATCC) were grown at the condition of 37°C with 5% CO_2_ after adding high glucose Dulbecco's modified Eagle's medium (DMEM) nutrient mix (10% FBS and 1% penicillin and streptomycin). Influenza A virus (IAV) (A/PR/8/34) was obtained from the Institute of Virology (Wuhan, China) and propagated in the allantoic cavity of 9‐day‐old embryonated eggs for 48 h at 37°C and then for 12 h at 4°C, after which allantoic fluid was collected. The hemagglutination titer of the virus in the allantoic fluid was detected by the hemagglutination method, and the allantoic fluid with a high hemagglutination titer was filtered and stored. Dilute the virus stock solution with DMEM (10^−1^, 10^−2^, 10^−3^, 10^−4^, and 10^−5^), add it to the A549 cells that grow into a monolayer, and incubate at 37°C for 2 h. At the end of the infection, the viral fluid was replaced by the maintenance medium containing DMEM, 100 U/mL penicillin and streptomycin, 1.2% BSA, and 1 µg/mL trypsin (used to activate the virus). After continued incubation for 72 h, the TCID50 (median tissue culture infective dose) of A/PR/8/34 was calculated by the Reed‐Muench method. A viral titer of 100 plaque‐forming units (PFUs) (PFU = 0.7 × TCID50) was used in subsequent in vitro experiments. All virus experiments were conducted in the Biosafety Level 2 laboratory of the Central Laboratory of Yichang Central People's Hospital.

### 2.2 Cell viability assay

The cytotoxic effects of AS‐IV (Solarbio, CAS:84687‐43‐4, HPLC ≥ 98%) on A549 cells were detected by Cell Counting Kit‐8 (CCK‐8, CK04). In brief, the A549 cells were seeded in 96‐well plates at a density of 1 × 10^4^ cells per well, and AS‐IV was diluted to the following concentrations: 1000, 500, 250, 125, 62.5, 31.2, and 0 μM added to the cells. After 72 h, 10‐μL CCK‐8 was added to each well immediately and continued to incubate for 2 h. The cell viability was calculated by reading the optical density (OD) value at 450 nm with a microplate reader (Thermo Fisher Scientific). The effect of AS‐IV on the viability of A549 cells infected with the influenza virus was also detected by the CCK‐8 method. A549 cells were seeded in 96‐well plates with partitions, and the experiment was set as the negative control group (NC), IAV group, and IAV + AS‐IV group. 2 h after virus infection, the cells were cleaned with phosphate buffer saline (PBS) solution twice, and then AS‐IV solution was added to IAV + AS‐IV group, and maintenance medium was added to the other groups. OD values were determined after continuous culture for 24 h.

### 2.3 Immunofluorescence staining

The 1 × 10^5^ A549 cells were seeded in 48‐well plates with partitions, and the experiment was set as NC group, IAV group, and IAV + AS‐IV group. 2 h after virus infection, the cells were cleaned with PBS solution twice, and then AS‐IV solution was added to IAV + AS‐IV group, and maintenance medium was added to the other groups. After a certain time, the cells were fixed with 4% paraformaldehyde for 30 min and then blocked with 1% bovine serum albumin + 0.2% TritonX‐100 for 1 h. Influenza A nucleoprotein (NP) antibody (Sino Biological, Cat: 11675‐MM03T; 1:200) was then added to the cells and incubated overnight at 4°C. The primary antibody was then absorbed and abandoned, fluorescein Dye488‐conjugated goat antimouse antibody was supplied by (Biqdoo‐Bio, B100812; 1:200) was added and incubated in the dark for 1 h, and finally Hoechst (Beyotime, C1022) was added to stain the nucleus for 15 min. The expression of NP was observed under the fluorescence microscope. Use Photoshop (2021) to merge images.

### 2.4 Measurement of total antioxidant capacity (TAC), superoxide dismutase (SOD), glutathione peroxidase (GPX), catalase (CAT), and malondialdehyde (MDA)

The 5 × 10^5^ A549 cells were seeded in 6‐well plates with partitions, the experiment was set as NC group, IAV group, and IAV + AS‐IV group, which were given corresponding treatment, and cells were collected 24 h later. According to the manufacturer's instructions, the levels of TAC (Beyotime, S0121), GPX (Beyotime, S0058), CAT (Beyotime, S0051), SOD (Beyotime, S0101S), and MDA (Beyotime, S0131S) were detected using the kits.

### 2.5 Flow cytometry

A549 cells were seeded in 6‐well plates with partitions, the experiment was set as NC group, IAV group, and IAV + AS‐IV group, which were given corresponding treatment for 24 h. The cells are then digested and collected with trypsin (GENOMBIO). The ROS assay kit (Beyotime, S0033S) was used to detect the intracellular total ROS levels. Fluorescence probe dichlorodihydrofluorescein diacetate (DCFH‐DA) was diluted in serum‐free medium at 1:1000 to a final concentration of 10 μmol/L. The cells were suspended in diluted DCFH‐DA at a cell concentration of 5 million/mL and incubated in a cell incubator at 37°C for 20 min. Invert and mix every 3–5 min so that the probe is in full contact with the cells. The cells were then washed three times with PBS to fully remove DCFH‐DA that had not entered the cells. DCFH‐DA was hydrolyzed by esterase in cells to produce DCFH, and DCFH was oxidized by ROS species to produce DCF with fluorescence. Finally, DCF was detected by a flow cytometer (CytoFLEX; Beckman). Because the fluorescence spectrum of DCF is very similar to that of fluorescein isothiocyanate (FITC), the parameter setting of FITC was used to detect DCF in this experiment.

### 2.6 Enzyme‐linked immunosorbent assay (ELISA)

After various treatments, the levels of IL‐1β (Beyotime, PI305; Meimian Industrial Co. Ltd., MM‐0040M2) and IL‐18 (Neobioscience, NOV‐NR‐E10256; Meimian Industrial Co. Ltd., MM‐0169M2) in the cell culture supernatant were detected by specific ELISA kits based on the instructions of the manufacturer. The value of absorbance was detected using an enzyme‐labeled instrument at 450 nm. The standard curve is made and the measured sample concentration is calculated according to the standard curve.

### 2.7 Quantitative reverse transcription‐polymerase chain reaction (RT‐qPCR)

After various treatments, the total RNA of each group of cells was extracted with trizol reagent and reverse‐transcribed according to the instructions of the reverse transcription PCR kit (Servicebio). Then, amplification was performed using qPCR synergrtic binding reagent Green Master Mix Reagent (Yeasen Biotechnology). The relative expression levels of messenger RiboNucleic acid (mRNA); in each sample were assessed using the 2^−ΔΔCt^ method and normalized to glyceraldehyde‐3‐phosphate dehydrogenase (GAPDH) expression. Primer design and synthesis are as follows:

**Table:** 

Sample	Primer	Sequence
A549 cells	NLRP3‐F	5′‐GAGGAAAAGGAAGGCCGACA‐3′
	NLRP3‐R	5′‐TGGCTGTTCACCAATCCATGA‐3′
	Caspase‐1‐F	5′‐AGACATCCCACAATGGGCTC‐3′
	Caspase‐1‐R	5′‐TGAAAATCGAACCTTGCGGAAA‐3′
	IL‐18‐F	5′‐ACTGTAGAGATAATGCACCCCG‐3′
	IL‐18‐R	5′‐AGTTACAGCCATACCTCTAGGC‐3′
	IL‐1β ‐F	5′‐GAGCAACAAGTGGTGTTCTCC‐3′
	IL‐1β‐R	5′‐ AACACGCAGGACAGGTACAG‐3′
	GAPDH ‐F	5′‐CGTGGAAGGACTCATGACCA‐3′
	GAPDH‐R	5′‐GGCAGGGATGATGTTCTGGA‐3′
Epithelial cells	IL‐18‐F	5′‐GACTCTTGCGTCAACTTCAAGG‐3′
	IL‐18‐R	5′‐CAGGCTGTCTTTTGTCAACGA‐3′
	IL‐1β ‐F	5′‐GGATGAGGACATGAGCACCT‐3′
	IL‐1β‐R	5′‐AGCTCATATGGGTCCGACAG‐3′
	GAPDH ‐F	5′‐CCTCGTCCCGTAGACAAAATG‐3′
	GAPDH‐R	5′‐TGAGGTCAATGAAGGGGTCGT‐3′

### 2.8 Western blot

After modeling, the total protein was extracted by radio immunoprecipitation assay buffer (Biosharp, BL504A), and protein concentrations were measured with a Bicinchoninic Protein Assay Kit (Biosharp, BL521A). The protein samples were analyzed in 10%–15% sodium dodecyl sulfate polyacrylamide gel electrophoresis with a voltage from 80 to 130 V, and then, transferred to 0.45‐μm polyvinylidene fluoride membranes (220 mA, 30–60 min). The protein bands were blocked with tris‐buffered saline containing 0.05% Tween‐20% and 5% skim milk for 1–3 h at room temperature and incubated overnight at 4°C with the following primary antibodies: GAPDH (CST, D16H11; 1:3000), NLRP3 (Abcam, AB 109414; 1:500), Pro‐Caspase‐1 (CST, D7F10; 1:1000), Cleaved Caspase‐1(CST, D57A2; 1:500). Next, wash off excess primary antibodies with tris buffered saline with tween (TBST). HRP‐conjugated secondary antibody (Servicebio, GB23303; 1:3000) was incubated with horseradish peroxidase conjugate at room temperature for 1 h. After washing the membranes again with TBST, the bands were analyzed with an ECL system (P10501; Applygen) and Image J software (Bio‐Rad).

### 2.9 Mice protection and lung lesion assay

#### 2.9.1 Model building

BALB/c mice (♂, ♀, 18–20 g) were purchased from the Animal Center of China Three Gorges University (Hubei, China). All mice were exposed to alternating light and dark for 12 h, and food and water were provided free of charge at the animal center.

The protective effect of AS‐IV on mice was observed by simulating IAV infection with poly (I:C) (Yuanye Bio‐Technology Co. Ltd., 24939‐03‐5) attack. The mice were randomly divided into NC, poly (I:C) group, poly (I:C) + AS‐IV (25 mg/kg) group, poly (I:C) + AS‐IV (50 mg/kg) group, and poly (I:C) + dexamethasone (DEX) (5 mg/kg) group (*n* = 6 for each group). Poly (I:C) group and drug therapy groups received poly (I:C) (100 μg/mice, dissolved in 50‐μL PBS) nasal drops for 3 days to establish pneumonia mice models, while the NC group received 50 μL of PBS nasal drops for 3 days. Then the mice were given different concentrations of AS‐IV, Dexamethasone (DEX, Solarbio, CAS: 50‐02‐2, purity >98%), or PBS, administered by intragastric administration every day for 5 days.

#### 2.9.2 Scoring and hematoxylin and eosin (H&E) staining of lung tissue

On the 6th day, the body weight of mice in each group was measured. Euthanasia was used to sacrifice mice, lung tissue was collected, and the lung weight of each mouse was measured. Lung index is expressed as the ratio of average lung weight to average body weight. After completing the above steps, the lung tissues of mice in each group were stained with H&E. Finally, observe under a microscope, collect images, and analyze. Pathological grading was performed according to the relative degree of lung injury and inflammatory infiltration.^26^ The scoring was on a scale from 0 to 4: 0, lung tissue structure is normal, no inflammatory cell infiltration; 1, mild structural abnormalities of lung tissue with or without inflammatory cell infiltration; 2, moderate abnormality of lung tissue structure, no inflammatory cell infiltration; 3, moderate abnormality of lung tissue structure with inflammatory cell infiltration; and 4, severe abnormality of lung tissue structure with or without inflammatory cell infiltration.

#### 2.9.3 Detection of IL‐1β and IL‐18 levels in mice lung epithelial cells

The lung tissue of mice was collected after the model was established according to the above steps 2.9.2. The lung tissue was cut into small pieces and rubbed repeatedly on a 70 μm cell sieve with a grinder. During this process, homogenate rinsing solution was continuously added, so that all the cells were flushed through the screen into the centrifuge tube. The tissue grinding liquid was centrifuged at 1500 r/min for 10 min, and the supernatant was discarded. The cell concentration was adjusted to 2 × 10^8^−1 × 10^9^/mL by resuspension with tissue sample diluent. Then, lung epithelial cells were isolated according to KIT instructions for mice tissue epithelial cell isolation solution (Haoyang Biotechnology, EP2014TBD). After isolation of mice lung epithelial cells, the contents of IL‐1β (Meimian Industrial Co. Ltd., MM‐0040M2) and IL‐18 (Meimian Industrial Co. Ltd., MM‐0169M2) in mice lung epithelial cells of each group were detected according to the instructions of Elisa kit. The levels of IL‐1β and IL‐18 in lung epithelial cells of mice in each group were detected by RT‐qPCR.

All operations and experiments were performed following the principles of the National Guidelines for Nursing.

### 2.10 Data analysis

All data were averaged from at least three independent experiments. All results were analyzed by SPSS 26.0 and reported as the mean ± standard deviation. Histograms were drawn using GraphPad Prism 9.4.0 software (San Diego, CA, USA). One‐way analysis of variance was used to test the differences between groups. *p* < .05 was considered as statistically significant.

## 3 RESULTS

### 3.1 AS‐IV increases the cell viability of influenza‐infected A549 cells but has no inhibitory effect on the influenza virus infection

**Figure 1:**
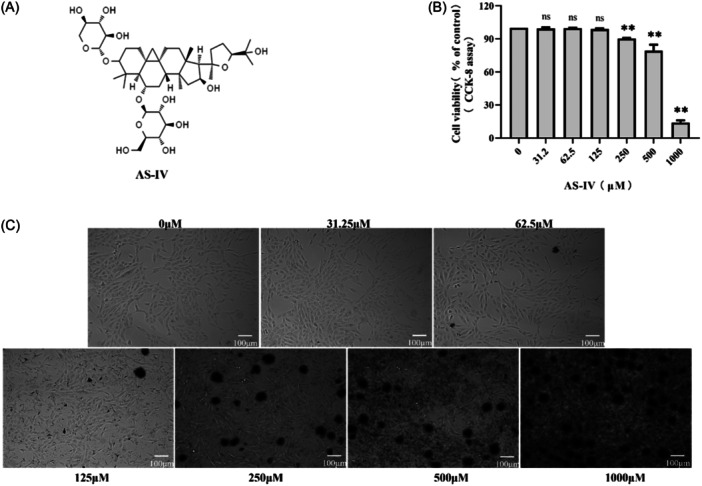
The cytotoxicity effects of astragaloside IV (AS‐IV) on the A549 cells. (A) Chemical structure of AS‐IV. (B) Cell Counting Kit‐8 (CCK‐8) reagent was used to detect A549 cell viability. Cell viability was defined as the percentage of the 0‐μM group (*n* = 3). (C) The growth of A549 cells was observed under the microscope (100x magnifying power). ns, no significant difference, ** *p* < 0.01, when compared to the 0 group.

Figure 1A shows the chemical structure of AS‐IV. Figure 1B,C shows the toxicity of A549 cells treated with AS‐IV for 72 h. The results showed that AS‐IV had no obvious toxicity to cells when the concentration reached 125 µM, but when the concentration reached 250 µM, AS‐IV formed many drug crystals, and the cell viability was significantly decreased (*p* < 0.01). Therefore, concentrations below 125 µM were used for subsequent experiments.

**Figure 2:**
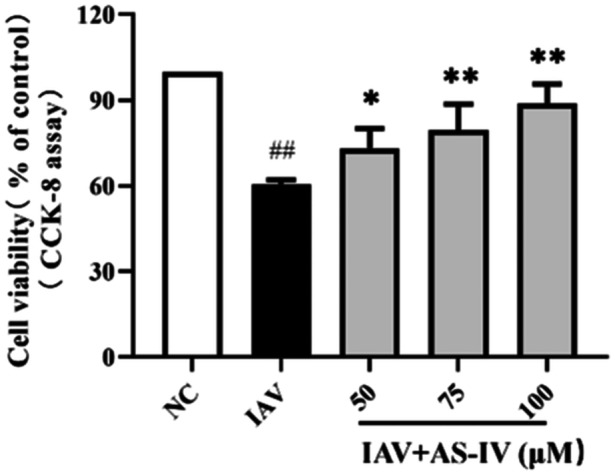
Protective effect of astragaloside IV (AS‐IV) on A549 cells infected with influenza virus. Cell Counting Kit‐8 (CCK‐8) reagent was used to detect A549 cell viability. Cell viability was defined as the percentage of the NC group (*n* = 3). NC, negative control group; IAV, influenza A virus. ^*##*^ *p* < 0.01, when compared to the NC group; * p < 0,05, ** p < 0.01, when compared to the IAV group.

A549 cells infected with the influenza virus (A/PR/8/34 at 100 PFU) were treated with 50‐, 75‐, and 100‐µM AS‐IV. After influenza virus infection, the cells showed swelling, rupture, and dissolution, and the cell density was significantly lower than that of the NC group, AS‐IV can significantly reduce the A549 cell death caused by viral infection (Figure 2).

**Figure 3:**
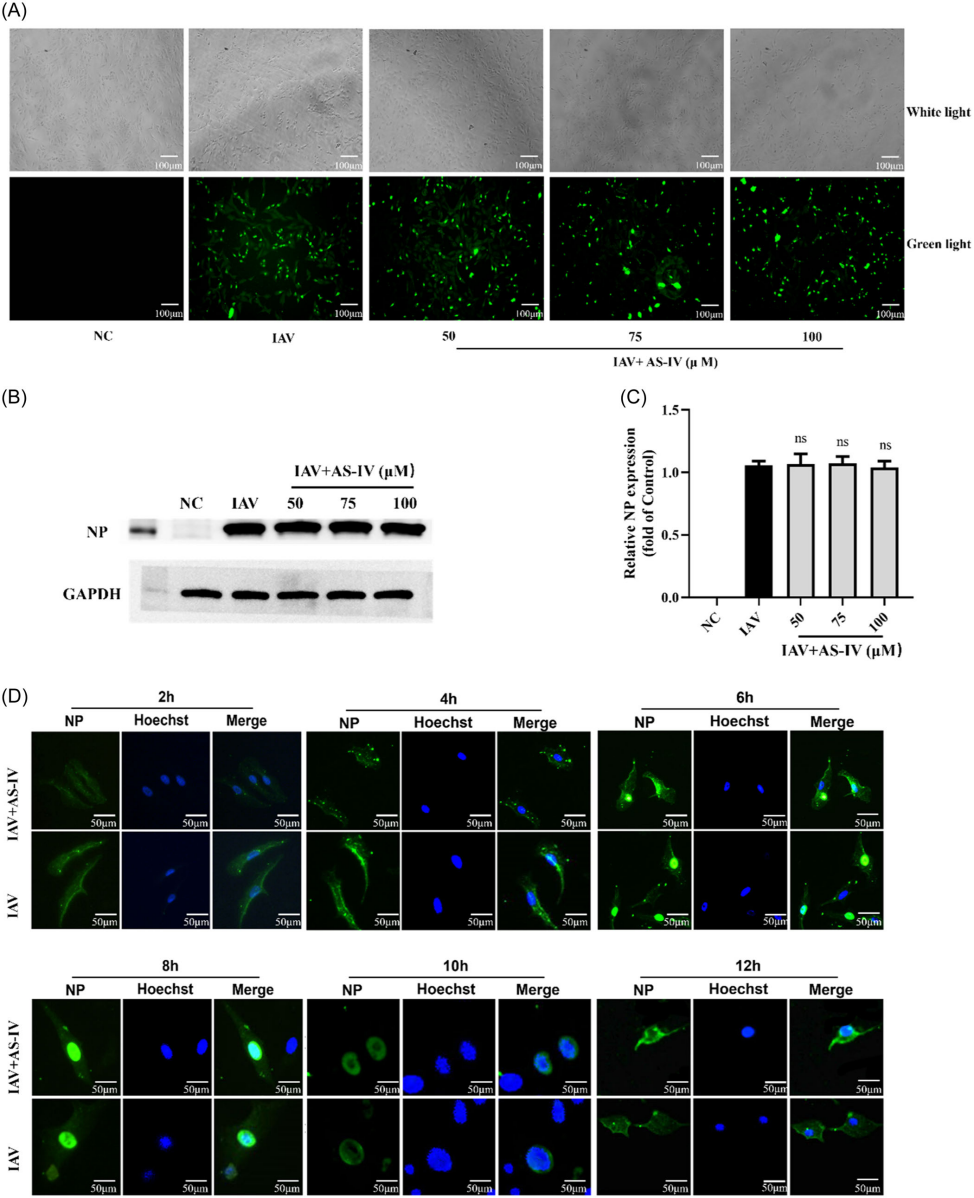
Effects of astragaloside IV (AS‐IV) on influenza virus in A549 cells. (A) A549 cells infected with influenza A virus (IAV) were treated with 50‐, 75‐, and 100‐μM AS‐IV, and the infection rate of IAV in A549 cells in each group was observed after 16 h (100x magnifying power). (B) and (C) Western blot detected NP protein expression in A549 cells treated with AS‐IV after influenza virus infection (*n* = 3). (D) 100‐μM AS‐IV treated A549 cells infected with IAV, and immunofluorescence was performed at different time points (2, 4, 6, 8, 10, and 12 h) to observe the effect of AS‐IV on nuclear membrane shuttle transport of IAV NP protein in A549 cells (200x magnifying power). ns, no significant difference, compared to the IAV group. NC, negative control group. NP, nucleoprotein.

To further evaluate the anti‐influenza virus effect of AS‐IV, we used the immunofluorescence method to evaluate the efficacy of AS‐IV on IAV. As shown in Figure 3A, AS‐IV had no obvious inhibitory effect on the infection of the influenza virus. AS‐IV also had no inhibitory effect on IAV replication (Figure 3B,C). In addition, we also observed that AS‐IV had no obvious effect on delaying the nuclear envelope shuttle transport of NP protein (Figure 3D).

### 3.2 AS‐IV downregulates ROS induced by influenza virus infection

**Figure 4:**
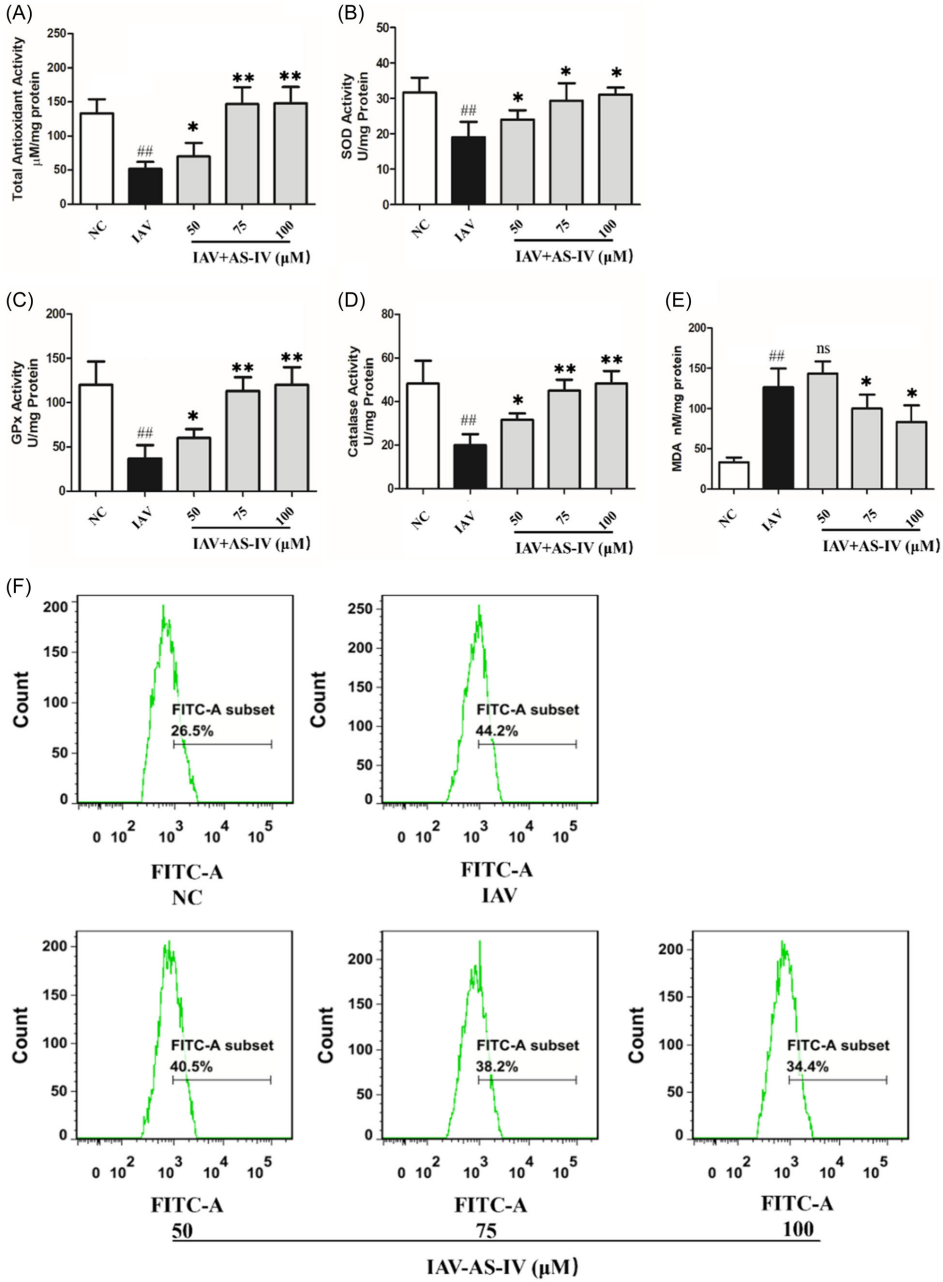
Antioxidative effect of astragaloside IV (AS‐IV). (A)–(D) Effects of AS‐IV on total antioxidant capacity, superoxide dismutase, glutathione peroxidase, catalase, and malondialdehyde in A549 cells infected with influenza virus (*n* = 3). (F) Effects of AS‐IV on reactive oxygen species in A549 cells infected with influenza virus. GPX, glutathione peroxidase; NC, negative control group; IAV, influenza A virus. ^*##*^ *p* < .01, when compared to the NC group; ns, no significant difference, * p < 0.05, ** p < 0.01, when compared to the IAV group.

To detect the antioxidant effects of AS‐IV, intracellular TAC, SOD, GPX, CAT, MDA, and ROS levels were measured. We confirmed that the levels of intracellular TAC, SOD, GPX, and CAT were significantly reduced after influenza virus infection, while AS‐IV treatment restored the inhibition of TAC, SOD, GPX, and CAT by influenza virus infection (Figure 4A–D). The results of MDA detection showed that MDA concentration in the IAV group was increased compared with the NC group, while MDA level in AS‐IV‐treated cells was downregulated compared with the IAV group (Figure 4E). As shown in flow cytometry analysis, the ROS levels were dramatically elevated after influenza virus infection, while the levels of ROS for AS‐IV groups were rescued after treatment (Figure 4F).

### 3.3 AS‐IV inhibits the activation of the NLRP3/Caspase‐1 signaling pathway induced by influenza infection

**Figure 5:**
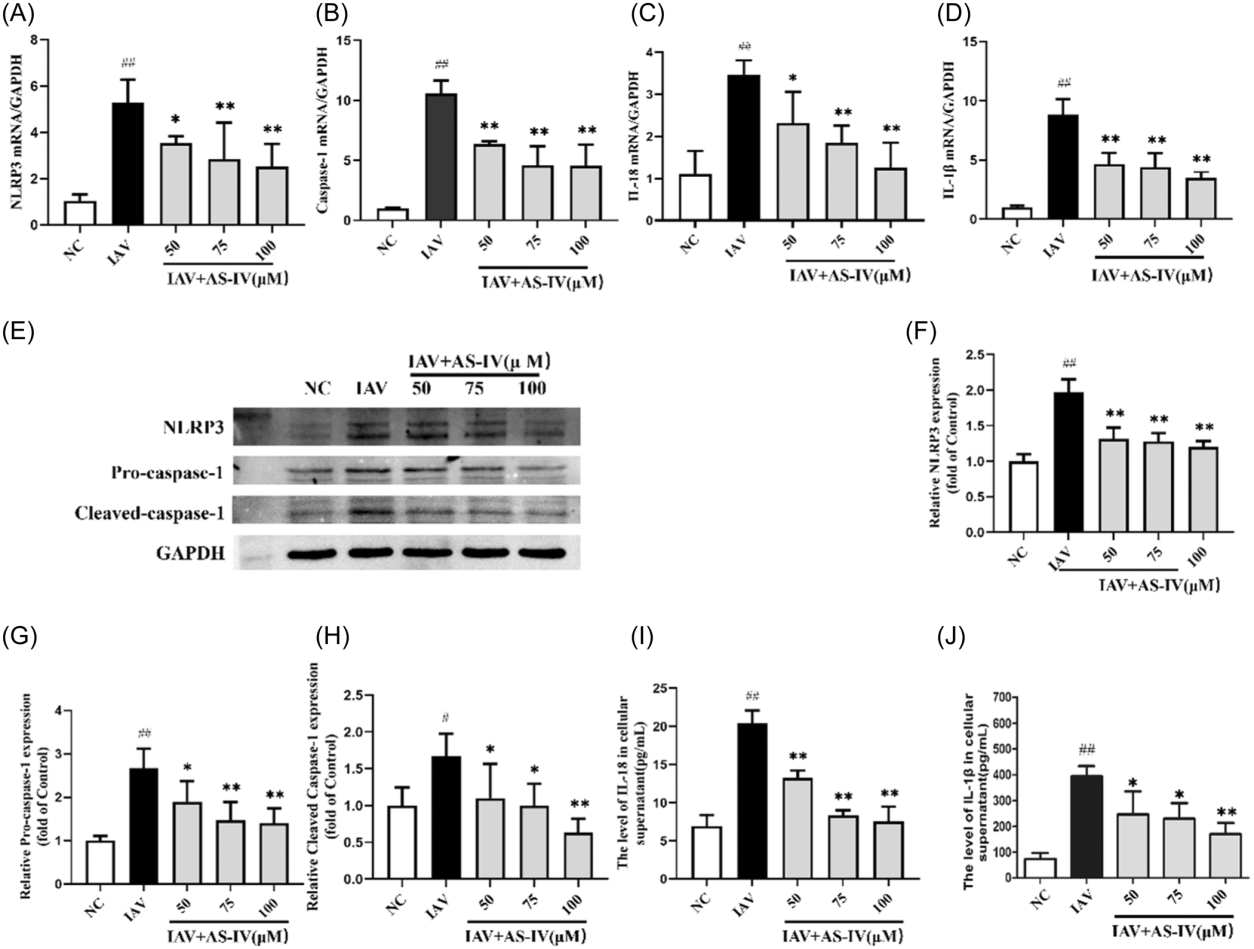
Effect of astragaloside IV (AS‐IV) therapy on the NOD‐like receptor thermal protein domain associated protein 3 (NLRP3) inflammasome pathway in A549 cells infected with influenza virus. (A)–(D) Real‐time polymerase chain reaction analysis was used to analyze the mRNA levels of NLRP3, Caspase‐1, IL‐1β, and IL‐18 in different groups. (E)–(H) The protein expression levels of NLRP3 inflammasome, Pro‐Caspase‐1, and Cleaved Caspase‐1 in each group were detected by western blot. (I)–(J) Enzyme‐linked immunosorbent assay was used to detect the expression levels of inflammatory cytokines IL‐18 and IL‐1β in each group. *n* = 3, NC, negative control group; IAV, influenza A virus. ^*#*^ *p* < 0.05, ^*##*^ *p* < 0.01, when compared to the NC group; * p < 0.05, ** p < 0.01, when compared to the IAV group. IL, interleukin; mRNA, messenger RiboNucleic acid; NOD, nucleotide‐binding oligomerization domain.

Former studies have shown that increased ROS can activate NLRP3 inflammasome‐mediated cell damage,^10^ so we examined the expression levels of factors associated with the NLRP3/Caspase‐1 signaling pathway in each group of cells. Our results showed that NLRP3 levels increased after influenza virus infection, but NLRP3 mRNA and protein levels decreased in a dose‐dependent manner after AS‐IV treatment (Figure 5A,E,F). Moreover, compared with the IAV group, the activation of Caspase‐1 in A549 cells after AS‐IV treatment and the secretion of cytokines IL‐18 and IL‐1β were decreased (Figure 5B–E,G–J).

### 3.4 AS‐IV attenuates lung inflammatory lesions in mice caused by poly (I:C)

**Figure 6:**
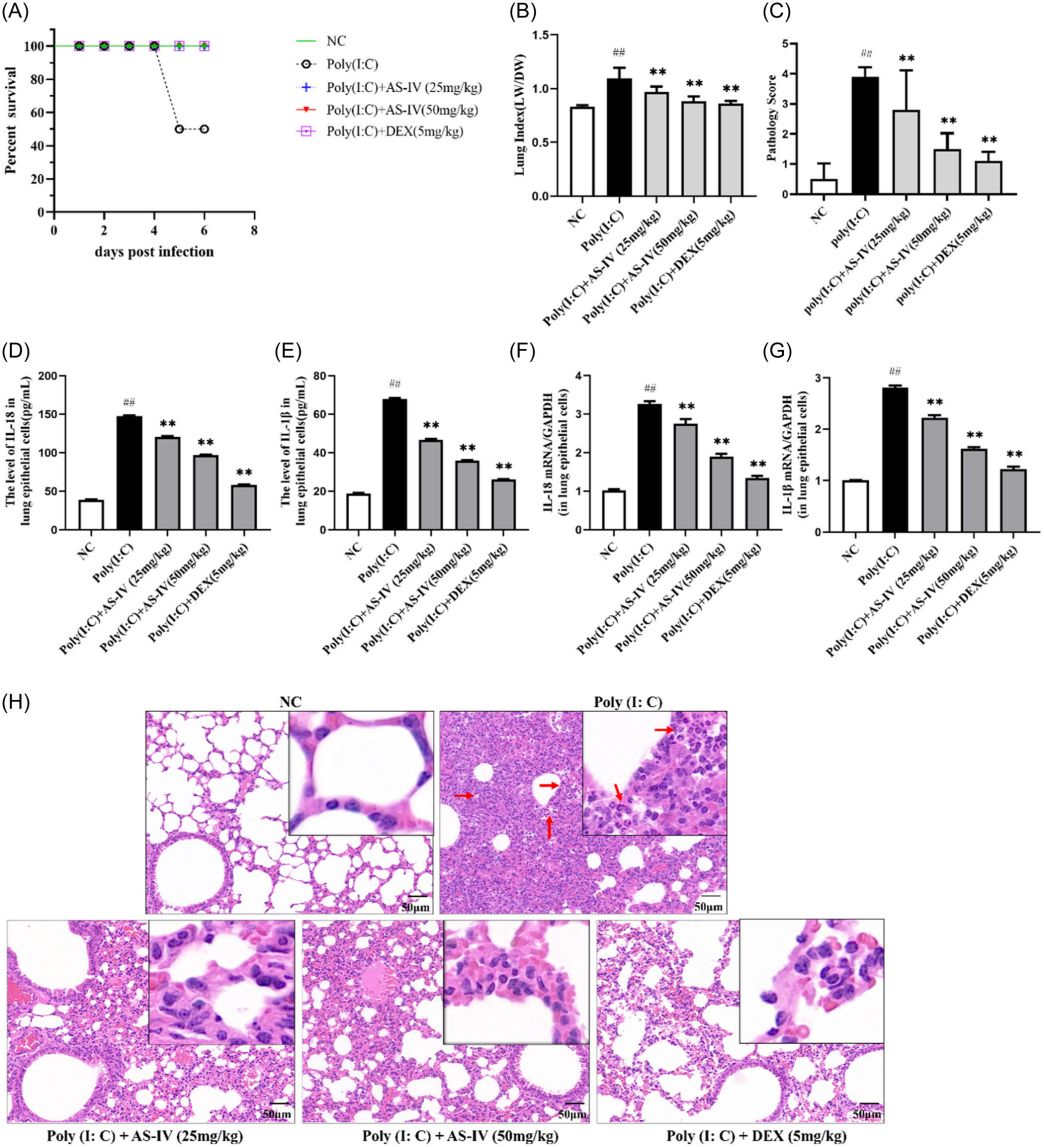
Effects of astragaloside IV (AS‐IV) treatment on poly (I:C)‐induced lung inflammatory lesions in mice. (A) Survival curve of each group of mice. (B) Lung index for mice killed on Day 6. (C) Pathological scores of lung tissue in mice of each group. (D) and (E) Enzyme‐linked immunosorbent assay detects levels of IL‐18 and IL‐1β in mice lung epithelial cells. (F) and (G) Quantitative reverse transcription‐polymerase chain reaction detects levels of IL‐18 and IL‐1β in mice lung epithelial cells. (H) Hematoxylin and eosin staining of lung tissue, the red arrow points to inflammatory cells (200x magnifying power). The number of samples in each group is *n* = 6. NC, negative control group. ^*##*^ *p* < 0.01, when compared to the NC group; * p < 0.05, ** p < 0.01, when compared to the poly (I: C) group. IL, interleukin.

The above results led us to further investigate whether AS‐IV can inhibit viral pneumonia induced by viral RNA mimics poly (I:C) in vivo. The state of the mice was observed daily. In the 5d modeling, it was observed that the vitality of the mice in group poly (I:C) was poor, the hair was rough, and one mouse died (Figure 6A). The status of the mice in the other treatment group was not significantly different from that in the normal group. Compared with the poly (I:C) group, the lung index and pathological score of the AS‐IV group were decreased (Figure 6B,C).

The results of ELISA showed that the levels of IL‐18 and IL‐1β in the lung epithelial cells of mice after poly (I:C) infection were significantly higher than those in the NC group(Figure 6D,E). After treatment with AS‐IV, the levels of IL‐18 and IL‐1β in lung epithelial cells of mice in the poly (I:C) + IAV group were significantly decreased compared with those in the poly (I:C) group. Meanwhile, PCR results further verified this result (Figure 6F,G).

H&E staining results showed that compared with the NC group, the lung tissue structure of mice in the poly (I:C) group was seriously abnormal, with a large number of alveolar epithelial cells proliferating and alveolar walls thickening, accompanied by a large number of inflammatory cells infiltrating. AS‐IV significantly alleviated the lung tissue structure destruction and inflammatory cell infiltration caused by poly (I:C) infection (Figure 6H).

## 4 DISCUSSION

Influenza virus infection has been a great challenge to the global health system, causing a large number of deaths and economic losses worldwide every year.^3^ The initial host response to influenza virus invasion is acute inflammation, one which is characterized by the activation of inflammatory cytokines or chemokines, leading to the recruitment of inflammatory cells.^27^ A moderate immune response will help clear the virus, while excessive immune stimulation can cause tissue and organ damage, and a large number of inflammatory cytokines will even overflow into the circulatory system, causing a systemic cytokine storm, resulting in multiple organ dysfunction.^28^, ^29^ This serious consequence urgently requires the development of drugs to reduce lung damage caused by the influenza virus, and the secondary development and application of existing drugs seem to be more advantageous.

Traditional Chinese herbs have been used to prevent and treat viral infections for hundreds of years and are popular around the world due to their good tolerance. AS‐IV is a natural compound with multitarget therapeutic properties extracted from *R. Astragali*, which has a good antiviral effect on hepatitis B virus,^30^ Coxsackie virus,^31^, ^32^ dengue virus,^33^ and so on. Its antiviral function has also been widely verified in respiratory diseases.^25^, ^34^, ^35^ Although Zhang et al. found that AS‐IV reduces the level of IL‐1β in influenza virus infection, the specific mechanism of action of AS‐IV in influenza virus infection remains unclear.^25^ Here, we further investigated the effect of AS‐IV on influenza virus infection and its underlying mechanism which may contribute to the application of AS‐IV and its analogs in the therapy of severe viral pneumonia.

ROS is a by‐product of biological aerobic metabolism and a general term for a class of oxygen‐containing and active substances. The balance of oxidative and antioxidant mechanisms is the key to maintaining ROS levels during physiological metabolism. Appropriate levels of intracellular ROS are necessary for signal transduction and apoptosis during cell growth, but excessive accumulation of ROS can cause oxidative stress leading to cell death.^36^, ^37^ After infecting the host, the influenza virus reduces and consumes the antioxidant oxidase activity of TAC, GPX, SOD, CAT, and other antioxidant systems, thereby increasing the ROS content in the body, leading to the occurrence of oxidative stress.^38^, ^39^, ^40^ Antioxidant enzymes can terminate free radical chain reactions, and MDA is the end product of free radical chain reactions and is widely used to measure the degree of oxidation deterioration in biological systems.^41^ Our results showed that compared with the NC group, influenza virus infection decreased the activities of TAC, SOD, GPX, and CAT in A549 cells, and significantly increased MDA and ROS levels, suggesting that influenza virus infection induced oxidative stress injury of A549 cells. After AS‐IV treatment TAC, GPX, SOD, and CAT were significantly increased, while MDA and ROS levels were significantly decreased, suggesting that AS‐IV has a strong antioxidant effect. These data indicate that AS‐IV also exhibits the ability to inhibit ROS and antioxidants in influenza virus infection. Our subsequent experiments showed that the mRNA and protein levels of NLRP3 were significantly increased in A549 cells after influenza virus infection, and its downstream Caspase‐1, IL‐18, and IL‐1β were also increased. When AS‐IV inhibited intracellular ROS levels in a dose‐dependent manner, intracellular NLRP3, Caspase‐1, IL‐18, and IL‐1β levels were all reduced in a dose‐dependent manner. Our data demonstrate that AS‐IV may inhibit influenza virus‐induced inflammation mediated by the ROS/NLRP3/Caspase‐1 signaling pathway in vitro.

In vivo, we selected poly (I:C) to simulate viral infection in mice. Poly (I:C) is a double‐stranded RNA analog that can cause lung inflammation, interstitial edema, bronchiolar epithelial hypertrophy, and lung function changes in mice, and can induce pro‐inflammatory factors IL‐6, TNF‐α, Elevated levels of IL‐1β and IL‐8 and increased inflammatory cells.^42^, ^43^ In our study, we found that after poly(I:C) stimulation, the vitality of the mice gradually deteriorated, the hair became rough, and even death occurred on the 5d of modeling, while the state of the AS‐IV treated mice was similar to that of the NC group mice, and no mice died. The observation of H&E staining results of lung tissue showed that the lung tissue structure of mice stimulated by poly(I:C) was severely damaged, accompanied by a large number of inflammatory cells infiltrated, and the pathological score and lung index of lung tissue were significantly higher than those of the NC group. In contrast, AS‐IV treatment significantly reduced the destruction of lung tissue structure by poly(I:C), and the pathological score and lung index of lung tissue were also decreased. We isolated lung epithelial cells from mice's lung tissue for verification. The results confirmed that poly(I:C) stimulation induced the production of a large number of inflammatory factors IL‐1β and IL‐8, and AS‐IV treatment effectively inhibited IL‐1β and IL‐8 secretion. These data illustrate that AS‐IV can effectively reduce the inflammatory response induced by poly(I:C) and reduce viral lung injury.

To be honest, this study has certain limitation. That is, in vivo, we only proved that AS‐IV can inhibit inflammation, and we did not use influenza virus infection model to further verify the effect of AS‐IV on ROS/NLRP3/Caspase‐1 signaling pathway. In the future work, we will further improve this part of content.

## 5 CONCLUSION

This study shows that AS‐IV can reduce the acute inflammatory response induced by influenza virus in vitro. The potential mechanism may be that AS‐IV inhibits the activation of NLRP3 inflammasome and reduces the secretion of inflammatory factors by inhibiting intracellular ROS levels after influenza virus infection. In addition, AS‐IV alleviates lung tissue structural damage caused by poly (I:C) and reduces lung inflammation in vivo. These data promote the possibility of AS‐IV in the future clinical treatment of viral pneumonia caused by influenza viruses. Our study also highlights antioxidants as one of the effective strategies to combat viral pneumonia.

## AUTHOR CONTRIBUTIONS

**Xiaoli Huang**: Methodology; Project administration; Resources; Software; Visualization; Writing—original draft. **Yifan Zhou**: Methodology; Project administration; Resources; Software; Visualization; Writing—original draft. **Yi Li**: Methodology; Project administration; Resources; Software; Visualization; Writing—original draft. **Ting Wang**: Investigation. **Yandong Chen**: Formal analysis; Supervision. **Yuanhong Zhou**: Data curation; Funding acquisition. **Xiaolin Zhou**: Conceptualization. **Qiang Liu**: Funding acquisition; validation; writing—review and editing. All authors have read and approved the manuscript.

## CONFLICT OF INTEREST STATEMENT

The authors declare no conflicts of interest.

## ETHICS STATEMENT

The China Three Gorges University approved the animal ethics permit for this study (SCXK 2022‐0061).

## Data Availability

Data for the results of this study are available from the corresponding author.
